# Metal Water-Sediment Interactions and Impacts on an Urban Ecosystem

**DOI:** 10.3390/ijerph14070722

**Published:** 2017-07-05

**Authors:** Lian Lundy, Luciana Alves, Michael Revitt, Dirk Wildeboer

**Affiliations:** Department of Natural Sciences, Faculty of Science and Technology, Middlesex University, The Burroughs, Hendon, London NW4 4BT, UK; L.Alves@mdx.ac.uk (L.A.); M.Revitt@mdx.ac.uk (M.R.); D.Wildeboer@mdx.ac.uk (D.W.)

**Keywords:** diffuse and point source pollution, urban receiving waters, urban sediment quality

## Abstract

The EU Water Framework Directive (WFD) requirement that all surface water bodies achieve good ecological status is still a goal for many regulatory authorities in England and Wales. This paper describes field and laboratory studies designed to identify metal contaminant loadings and their distributions within water bodies located in the Lower Lee catchment (London, UK). Water and sediment samples have been collected from increasingly urbanised sites on the River Lee and its main tributaries over a two-year period with samples analysed for total concentrations of cadmium, copper, lead, mercury, nickel, tin, and zinc. Complimentary batch tests indicate a positive relationship between aqueous metal concentrations and the batch test-derived sediment metal release data, particularly during wet weather events. Field data indicate a dynamic relationship between water and sediment concentrations with both being capable of exceeding relevant environmental quality standards/sediment quality guidelines at all sites. Mean sediment metal concentrations across all sites were found to be highest for Cu (141.1 ± 111.0 µg g^−1^), Pb (175.7 ± 83.0 µg g^−1^), and Zn (499.9 ± 264.7 µg g^−1^) with Zn demonstrating elevated mean water concentrations (17.2 ± 13.8 µg L^−1^) followed by Ni (15.6 ± 11.4 µg L^−1^) and Cu (11.1 ± 17.8 µg L^−1^).

## 1. Introduction

The European Union (EU) Water Framework Directive [1] established a framework for water protection and management with the objective that surface and ground waters in all member states should achieve good ecological and chemical status by 2015 followed by two six-year cycles to allow further development of river basin management plans. A total of 45 substances, including five metals, have been identified as being of particular concern with 24 of them (including Ni and Pb) classified as priority substances (PS) and 21 designated as priority hazardous substances (PHS; including Cd, Hg and Sn (as tributyltin)) [2]. Pollutants classified as PS were required to meet Environmental Quality Standards (EQS) by 2015 with a PHS designation signifying that emissions of these substances to water need to cease. However, in 2015 within the Thames river basin district, which incorporates the River Lee catchment, it was reported that 45% and 17% of water bodies continued to be affected by point pollution discharges of wastewater and urban diffuse pollution, respectively [3]. 

The Lower Lee catchment has a long history of water quality problems as a consequence of increasing urbanisation associated with factors, such as discharges from both point sources (wastewater treatment plants (WWTPs)), industry, commercial enterprises), diffuse sources (e.g., runoff from roads and associated urban surfaces), navigation, and water abstraction [4,5]. Wastewater discharges have been heavily implicated in the failures of surface water quality to comply with EQS with, for example, 34% of Ni originating from domestic sewage, compared with 25% from runoff sources [6]. This difference has the potential to be greater when industrial wastewaters are discharged to municipal WWTPs.

In polluted waters, many contaminants are predominantly adsorbed to suspended particles in the water column and to sediments settled on the river bed. This identifies sediments not only as pollutant sinks but also as potential sources of contamination as a result of changes in environmental conditions and/or anthropogenic disturbances. Sediments represent a more stable medium for tracing metal sources compared to water [7]. Whereas aqueous phase sampling provides an indication of metal concentrations on a relatively short time scale not exceeding hours, sediments can be representative of pollutant trends over longer periods, usually up to one year [7,8,9]. For this reason, bed sediments have been increasingly employed in the assessment of the contamination of fluvial systems in urban and suburban areas [10,11,12,13,14]. 

Sediment quality criteria are not specifically identified in the existing legislation associated with the Water Framework Directive [2], but their implications for water quality and ecosystem health are recognised [15] together with their relevance to long-term pollutant accumulation [16]. Contaminants in sediments may be mobilised by river processes (e.g., storm events, influx from groundwater and bio-turbation) or human activities (e.g., dredging, permitted and unpermitted discharges of effluents and runoff, and recreational activities), which can cause release to the overlying water column and the potential for downstream transport [17]. The spatial variability of metal content in river water and sediment depends on the ability of geochemical factors [18] and chemical parameters such as pH, dissolved organic carbon (DOC) and redox potential [19] to influence sediment-water interactions. Tackling the risks posed by contaminated sediment represents a significant challenge which has implications for the interaction between land-based activities, management of water and protection of the wider environment and human health [20]. For these reasons, sediment EQS have been developed by individual countries, including Canada [21], The Netherlands [22] and France [23]. Duodu et al. [24] have proposed the use of a range of sediment quality indices, including the contamination factor, enrichment factor, index of geo-accumulation, modified degree of contamination, pollution index, and modified pollution index to comprehensively ascertain the sediment quality.

Rivers represent key resources in terms of providing water for drinking supply, industry, and crop cultivation. Additionally, in urban catchments they are required to receive wastewater and surface runoff discharges whilst maintaining recreational and conservation facilities. This paper investigates the ability of the lower River Lee, an important urban river in North East London, to fulfil these functions through a study of the spatial and temporal trends in the metal levels in both sediments and the overlying waters. Seven metals (Cd, Cu, Pb, Hg, Ni, Sn, and Zn) have been monitored to obtain a better understanding of how sediment-water interactions can influence their distribution and hence their impact on the urban river environment.

## 2. Materials and Methods

### 2.1. Sampling Sites and Sample Collection

The waterways within the Lower Lee catchment serve as sources of water supply for London, recipients for treated sewage discharges, navigation channels, as well as providing recreational and environmental resources. The catchment drains an area of 367.4 km^2^ in which the geology consists of London clay with deposits of alluvium and river gravels, overlying chalk. Consequently, the river levels can peak rapidly during high rainfall events leading to the potential for flooding. 

**Figure 1:**
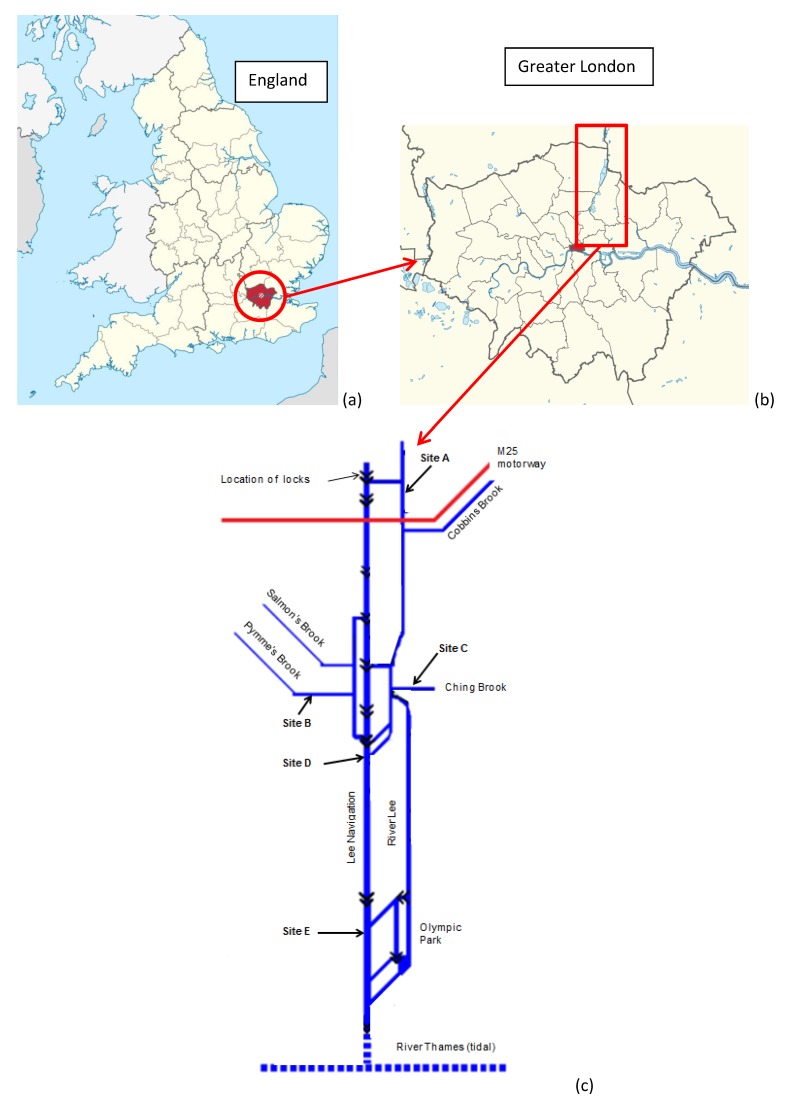
Location maps (**a**,**b**) and schematic identifying location of sampling sites on the Lower River Lee (**c**).

Water and sediment samples were collected from five progressively urbanising sites (labelled A to E; see Table 1 and Figure 1) on eight occasions between November 2014 and March 2016. An Ekman grab was used for sediment collection and surface water samples were obtained using a polypropylene dipper fitted with a 500 mL cup. Collected water samples were immediately transferred to 500 mL acid-washed plastic bottles and stored in ice during transfer to the laboratory and storage at 4 °C prior to preparation for analysis. Surficial bottom sediments were returned to the laboratory on ice for oven drying overnight at 105 °C. Prior to extraction the dried samples were gently ground and sieved to particle sizes ≤1 mm. In association with sample collection, in situ measurements of temperature, dissolved oxygen (DO) and pH were obtained.

### 2.2. Batch Tests

The procedure for the batch tests was adapted from that described by the Organisation for Economic Co-operation and Development (OECD) guidelines [25]. The tests were conducted in duplicate, for each of the five monitored sites, in 1 L sealed and light-excluded sterile bottles using a ratio of sediment (30 g dry weight) to river water (900 mL). After an initial stabilisation period of 24 h, stirring was initiated using polytetrafluoroethylene (PTFE) coated magnetic stirring bars. After stirring for 24 h a 2 h period of settling was allowed. Subjecting the supernatant to a centrifugal force of 3060× *g* for 10 min provided water samples for analysis as described in Section 2.3. Measurements of DO, pH and temperature in the water column were taken throughout the batch tests.

### 2.3. Laboratory Analysis

Water and sediment samples were subjected to microwave digestion (MARS press; CEM Corporation, Matthews, USA) according to an adaptation of US EPA methods 3015A [26] and 3051A [27]. Water samples (27 mL) were placed in pre-cleaned PTFE-TFM digestion vessels followed by the addition of concentrated (70%) nitric acid (2 mL) and concentrated hydrochloric acid (1 mL). Deionised water (27 mL) was used as the reagent blank. The vessels were sealed and after microwave digestion each water sample was filtered through Whatman ash-less, grade 42 filter papers and diluted to 50 mL using deionised water. All glassware was soaked overnight in 10% nitric acid, rinsed with deionised water, and oven dried prior to use. Extracted water samples were analysed for metals using ICP-MS (X-Series 2, Thermo Fisher Scientific, Hemel Hempstead, UK). Instrument detection limits, calculated as three times the standard deviation of the blank, were 9.2 ng L^−1^, 29.4 ng L^−1^, 223.7 ng L^−1^, 75.1 ng L^−1^, 7.3 ng L^−1^, 22.9 ng L^−1^, and 47.7 ng L^−1^ for Cd, Cu, Hg, Ni, Pb, Sn, and Zn.

Sediment samples (accurately weighed to approximately 0.5 g) were extracted in pre-cleaned PTFE-TFM digestion vessels using concentrated (70%) nitric acid (9 mL) and concentrated hydrochloric acid (3 mL). An analytical reagent blank was prepared using the acids only. After microwave digestion and subjecting to a centrifugal force of 3060× *g* for 10 min, the supernatant was transferred to a volumetric flask, and together with washings, made-up to a volume of 100 mL with deionised water. The metal concentrations of all sediment samples were determined using ICP-OES (iCAP 6000, Thermo Fisher Scientific). Instrument detection limits were 0.3 ng g^−1^, 0.8 ng g^−1^, 0.8 ng g^−1^, 0.4 ng g^−1^, 1.0 ng g^−1^, 1.3 ng g^−1^, and 0.2 ng g^−1^ for Cd, Cu, Hg, Ni, Pb, Sn, and Zn. Using a certified reference material (SQC001 (Lot 011233), Sigma-Aldrich, Poole, UK), recovery efficiencies from sediment samples of between 81.1% for Ni and 96.5% for Zn were determined. Multi-element calibration standards of 0.1, 0.5, 0.75, and 1 mg L^−1^ were prepared for sediment analyses with the six standards for water analyses covering the concentration range of 2–20 µg L^−1^. All analyses were carried out in triplicate and daily performance checks were conducted on each instrument prior to commencement of analyses. The instrument calibrations were checked every 10 samples throughout the analytical procedure by running one of the calibration solutions as an unknown. Recalibration was performed if drifts in the calibration measurements exceeded 10%. The precision of the methodology (% RSD) for water extractions was typically <15% for all metals, but improved to < 5% for sediment extractions, except for Sn where it was <10%.

## 3. Results and Discussion

### 3.1. Concentrations of Selected Metals and Associated Parameters in Surface Waters

The concentrations of selected metals in surface waters collected at the five sites over a two year period are shown in Table 2. The monitored ranges at each site show considerable variations (generally 1–2 orders of magnitude, but up to three orders of magnitude for Sn at site E), which are characteristic of sites receiving point and diffuse pollution inputs [28,29]. In comparison with an earlier study of metal pollution within the River Lee [4], mean aqueous concentrations of Cd, Cu, Ni, Zn and Pb, are generally lower and it may be that on-going pollution mitigation measures, such as modernisation of WWTPs, installation of sustainable drainage systems (SuDS), and sediment dredging have resulted in a decline in surface water metal concentrations. However, the earlier study reported comparatively lower Hg and Ni concentrations, suggesting specific sources of these metals have yet to be remediated. Despite the currently-observed variabilities in metal concentrations, some trends are apparent. For example, the mean and maximum metal concentrations determined in the two tributaries, site C (mixed industrial-urban land use) and, particularly, site B (densely urbanised land use) (see Figure 1), consistently exceed the concentrations at the upstream site A (with the exception of Hg and Sn). The tributaries are shown to be important contributors of metals to the main River Lee leading to elevated mean concentrations at the highly-urbanised downstream site E. Within the main channel (sites A, D, and E), mean concentrations of Cd and Pb increase in a downstream direction as would be expected due to progressively increasing urbanisation (see Table 2). However, other metals were not consistent with this trend with the highest mean concentrations of Hg being observed at site A, Cu, Ni, and Zn at site D, and Sn at site E, indicating that the different sites are in receipt of multiple sources of pollutants. The elevated concentrations determined at site D are associated with the fact that it is located at the point where a WWTP discharges treated effluents into the River Lee. 

Two of the eight sampling collections occurred following wet weather conditions (defined as rainfall ≥4 mm within the previous 48 h). The highest mean concentrations of Zn, Ni and Sn (with the exception of site A for Sn) were monitored on these occasions (May 2015 (4 mm) and August 2015 (15.2 mm)). In contrast the highest mean concentrations of Cd (except at Site A) and Cu were consistently reported during a dry weather sampling event (March 2016; no rainfall within the previous 48 h) indicating that the catchment is in receipt of pollutants during both dry (point source) and wet weather (diffuse) events and that some pollutants may be preferentially associated with specific sources. These results highlight the complexities associated with identifying pollutant sources in an urban catchment [5,30,31,32].

EU environmental quality standards (EQS) are available for Cd, Hg Ni, and Pb, and UK WFD Technical Advisory Group (TAG) standards [33] exist for Cu and Zn (see Table 2). A comparison of the determined concentrations with these standards indicate that mean concentrations of Pb and Ni exceed the annual average (AA) EQS at all sites with the maximum concentrations of Cd exceeding the AA value at sites B, C, D and E. Exceedance of the AA value indicates that there is a chronic threat to receiving water status. Metal concentrations greater than the maximum allowable concentration (MAC) are indicative of a short-term or acute risk to receiving water health. Mean concentrations of Hg exceed the corresponding MAC values at sites A, B, D, and E with maximum concentrations of Ni mirroring this behaviour at sites B and D. Mean concentrations of Cu and Zn exceed the appropriate UK TAG standards [33] at all sites (apart from Zn at site A) identifying a negative impact to the ecological status of these sites under acute and chronic scenarios. DO, pH, and temperature measurements were taken at each site at the time of sampling. Mean values over the two year monitoring period ranged from 7.2 to 8.9 mg L^−1^ (for DO), from 8.1 to 8.7 (for pH) and from 12.9 to 15 °C (for temperature), with no parameter showing a consistent trend by site location or sampling date (data not presented). Comparisons with UK TAG water quality guidelines [33] for rivers indicate that samples fall under the high category for DO and the ‘high/good quality’ for pH.

### 3.2. Concentrations of Selected Metals in Surficial Sediments

An overview of the concentrations of selected metals in surficial sediment at each of the five sites is reported in Table 3. As with surface water metal concentrations, sediment metal concentrations show considerable variation between and within sites, generally within an order of magnitude, but with concentrations of Ni and Hg varying across three orders of magnitude on some occasions. With the exception of Ni, mean sediment metal concentrations are consistently lowest at the upstream site (site A) and Cd and Hg show the expected increase through the main river channel (i.e., site A < D < E). In contrast, mean sediment concentrations of Cu, Pb, Sn, and Zn are highest at site D, probably due to its location downstream of both the polluted Site B, which has been identified as contributing metals to the system) and a WWTP. 

In the absence of national or international environmental quality standards for sediments, Table 3 lists the available Dutch and Canadian sediment quality guidelines. A comparison of mean concentrations with these values indicates that Cu, Zn, Pb, and Hg exceed the Dutch target values (TV) and the Canadian interim sediment quality guideline (ISQG) value at all sites (with the exception of Site A for Pb and Hg in relation to the TV). Exceedance of these values indicates sediments will be unable to fully recover their functional properties (TV) and that biological impacts are expected to occur (ISQG). A comparison with the Dutch intervention value (IV; concentrations at which functional sediment properties are seriously impaired) and Canadian probable effects level (PEL; the level above which biological effects are expected to occur frequently) indicate that Cu and Zn exceed the IV at site D only, with all metals (except Ni) exceeding PEL values at least at one site. Four metals exceed at least one set of guideline values at sites D (Cu, Hg, Pb, Zn) and E (Cd, Hg, Pb and Zn), indicating elevated levels of contamination, and these are, therefore, highlighted as key pollutant sinks within the system. 

### 3.3. Release of Sediment Metals to Overlying Waters during Laboratory Batch Tests

Table 4 identifies the amount of metal released into the overlying water column during the batch experiment (expressed as the percentage mass of metal released into solution compared to the total amount of metal contained in the sediment). A positive metal release into overlying water occurs for sediments collected from all five sites. Although the continual stirring associated with the batch test experiments does not directly simulate the conditions encountered within the field, the results confirm the potential for sediments to release metals and highlight the need for further investigation of the influencing conditions. This is particularly important with respect to the development and implementation of programmes of measures to achieve good ecological status under the EU WFD [1,34,35]. Whilst the amount of metal released into the sediment varies between metals and sites, the level of variation is generally within an order of magnitude, ranging from a low of 0.12% (Sn: site A) to a maximum of 6.12% (Cd: site B). Reported levels of metal release are greatest for Cd (3.33–6.12%) and Zn (3.12–4.73%) which is consistent with studies in the literature that identify Cd and Zn as pollutants which typically associate most readily with the dissolved phase [36,37]. 

All metals (with the exception of Hg) show the greatest release at site B (*r* = 0.59; *p* ≤ 0.05) indicating that metals stored in sediments at this site are potentially more susceptible to release. Greater potential for the release of metals from sediments at this site could also indicate that sediments are a source of the elevated aqueous metal concentrations also reported at site B (see Section 3.1). Correlation analysis of sediment metal release data with levels of pH and dissolved organic content (DOC) determined after the experiment does not suggest that any of these parameters are responsible for the variations in metal release reported from sediments except for Pb and Ni where an inverse correlation with pH (*p* ≤ 0.05; *r* = −0.9) exists. Further examination of the sediments regarding their mineral and total organic content composition, sediment pH and cation exchange capacity may reveal the processes driving this relatively higher release for other metals at this site, but these were not within the scope of the current study.

### 3.4. Integration of Aqueous and Sediment Metal Concentration Data with the Results of Laboratory Batch Experiments

The reported results (Table 2 and Table 3) confirm the expected ability of the urban river sediments at all study sites to concentrate metals relative to the overlying waters [38,39,40]. However, the metal concentration trends do not necessarily parallel each other in the different phases. Thus, in this study, water samples indicated the highest mean metal concentrations at the two tributary sites: Pymmes Brook (Site B: Pb, Ni and Zn) and Cobbins Brook (Site C: Cd, Cu, Hg and Sn). In contrast, the highest mean sediment metal concentrations were found on the main river at Site D (Cu, Pb, Sn Zn) and Site E (Cd, Hg, Ni). Whilst the data ranges reported for sediment and aqueous concentrations varied between sampling dates and sample sites (as indicated by the magnitude of the associated standard deviations), sediment concentration variability was considerably less supporting the use of sediments as a better indicator of, and hence a preferred medium for, monitoring the environmental quality of aquatic systems [7,8,41]. 

**Figure 2:**
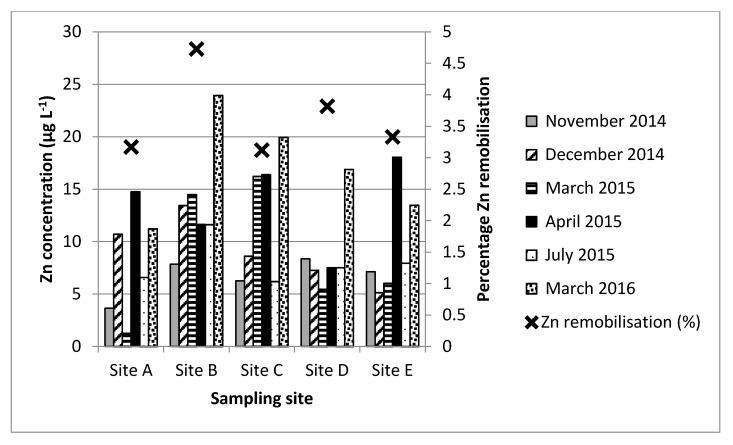
Overview of mean Zn aqueous concentrations during dry weather events at sites A–E in relation to Zn sediment remobilisation (%) determined during laboratory batch tests.

**Figure 3:**
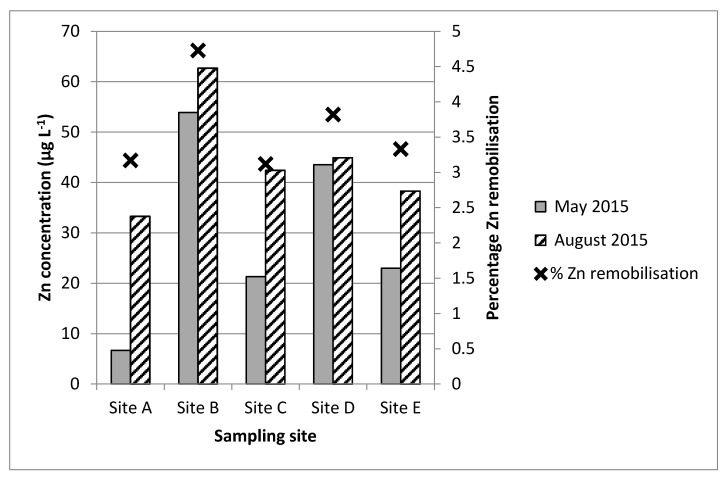
Overview of mean Zn aqueous concentrations during wet weather events at sites A–E in relation to Zn sediment remobilisation (%) determined during laboratory batch tests.

Analysis of the mean water and sediment metal concentrations did not reveal a statistically significant relationship between the two environmental compartments. This demonstrates the complex relationship that exists between metal behaviours within the two matrices. However, whilst mean aqueous metal concentrations did not appear to vary significantly in relation to mean sediment metal concentrations (see Section 3.3), the correlation analysis revealed a relationship between aqueous metal concentrations and the batch test-derived sediment metal release data, particularly during the two wet weather events. For example, aqueous Zn concentrations correlate with the sediment metal release data during both wet weather events (May 2015 and August 2015; *r* = 0.910 and 0.938; respectively; *p* ≤ 0.05 (both values)); see Figure 2 for dry weather data and Figure 3 for wet weather data). 

Likewise, positive significant correlations were also evident during the August wet weather event for Cu (*r* = 0.928; *p* ≤ 0.05) and Pb (*r* = 0.875; *p* ≤ 0.05), and for Hg and Sn during the May wet weather event (*r* = 0.93 and 0.87; *p* ≤ 0.05 both values, respectively). Significant positive correlations were also determined during some of the dry weather events (July 2015; Hg, Zn, Ni), November 2014 (Hg), December 2014 (Cd), and March 2016 (Hg). Whilst the overall pattern is not clear or consistent for all metals, the data does support the suggestion that the aqueous metal water concentrations are influenced by the releasable sediment metal concentrations (as determined from batch tests) during both wet and dry weather conditions in the field. The enhanced flow volumes and flow rates associated with wet weather conditions will encourage the resuspension of sediment and, consequently, the release of previously particulate-associated metals [42], as has been demonstrated in this study under laboratory conditions. The occurrence of positive correlations between aqueous metal and releasable sediment metal concentrations during dry weather could indicate the involvement of other sources of sediment resuspension activities, e.g., accidental spills or localised events, such as disturbance caused by human activities. 

## 4. Conclusions

Analysis of water and sediment metal concentrations showed that tributaries originating from heavily urbanised environments contribute to the heavy metal load of the River Lee. Metals transported into the river are likely to be stored in the sediment and, as shown by the batch tests, can be released into the overlying water, resulting in exceedances of water quality standards. The proportions of metals released varied with different weather conditions and site specific parameters. Zinc and Hg were found to be released most frequently, based on the correlation between sediment release potential and water concentrations, with concentrations of Zn consistently exceeding the UK TAG standards during correlated events and are, therefore, of particular concern. The results of this study highlight the importance of monitoring sediment as well as water quality and further analysis of sediment properties, water parameters, and chemical speciation of metals can strengthen the capacity of predicting pollutant release from contaminated sediments into the overlying water body. The findings are of relevance to all urban rivers in receipt of point and diffuse pollution, and of particular pertinence to those requiring remediation to achieve the environmental objectives of the EU Water Framework Directive [1]. 

## Figures and Tables

**Table 1:** Location of sampling sites in the Lower Lee catchment.

Site	Description of Sampling Site Location
A	Upstream site on River Lee on northern edge of London boundary and above the M25 orbital motorway
B	On the Pymmes Brook before its confluence with the River Lee; approaching from a westerly direction
C	On the Ching Brook before its confluence with the River Lee; approaching from an easterly direction
D	On the River Lee before separation into the Lee Navigation Channel; at a mid-point down the Lower Lee Catchment
E	On the lower reaches of the River Lee prior to its confluence with the River Thames

**Table 2:** Overview of the range of concentrations (minimum-maximum) and mean (µg L^−1^ ± SD) total metal concentrations in surface waters determined at five sampling sites together with relevant water quality standards.

Site	Cd		Cu		Hg		Ni		Pb		Sn		Zn	
	Range	Mean ± SD	Range	Mean ± SD	Range	Mean ± SD	Range	Mean ± SD	Range	Mean ± SD	Range	Mean ± SD	Range	Mean ± SD
A	0.02–0.15	0.07 ± 0.10	1.04–27.2	7.73 ± 10.3	0.02–1.00	0.35 ± 0.40	0.66–25.9	9.73 ± 7.90	0.21–3.93	1.54 ± 1.40	0.10–2.38	0.65 ± 0.90	1.25–33.3	11.0 ± 9.90
***B***	***0.04–0.44***	***0.14*** ***±*** ***0.10***	***1.06–28.2***	***10.4*** ***±*** ***10.7***	***0.01–0.41***	***0.10*** ***±*** ***0.10***	***7.37–55.1***	***23.41*** ***±*** ***18.8***	***0.92–13.3***	***3.75*** ***±*** ***4.10***	***0.07–1.86***	***0.59*** ***±*** ***0.60***	***7.85–62.7***	***24.9*** ***±*** ***21.2***
***C***	***0.01–0.35***	***0.09*** ***±*** ***0.10***	***1.73–32.5***	***9.68*** ***±*** ***11.1***	***0.01–0.20***	***0.06*** ***±*** ***0.10***	***5.89–28.9***	***15.4*** ***±*** ***8.20***	***0.72–4.87***	***1.87*** ***±*** ***1.40***	***0.09–1.02***	***0.34*** ***±*** ***0.30***	***6.18–42.4***	***17.2*** ***±*** ***11.8***
D	0.01–0.33	0.08 ± 0.10	1.21–46.7	10.14 ± 16.0	0.01–0.29	0.08 ± 0.10	5.25–44.6	16.22 ± 14.4	0.51–3.40	1.68 ± 0.90	0.09–1.91	0.58 ± 0.70	5.45–44.9	17.7 ± 16.7
E	0.01–0.39	0.15 ± 0.10	1.92–27.0	8.73 ± 9.90	0.01–0.40	0.13 ± 0.20	4.81–24.2	12.96 ± 8.10	0.33–4.71	2.20 ± 1.40	0.06–13.9	2.15 ± 4.80	5.12–38.3	14.9 ± 11.4
UK TAG			1.00 ^(a)^										14.2 ^(b)^	
EQS AA *	0.25 ^(c)^						4.00 ^(a)^		1.20 ^(a)^					
EQS MAC **	1.50 ^(c)^				0.07 ^(d)^		34.0		14.0					

Key: * Annual average; ** Maximum allowable concentration; ^(a)^ Bioavailable fraction; ^(b)^ Bioavailable fraction 10.90 μg L^−1^ + Ambient background concentration 3.3 μg L^−1^ dissolved Zn for River Lee; ^(c)^ For Cd and its compounds the EQS values here are for Class 5 (≥200 mg CaCO_3_ L^−1^) as per hardness of water in the Lower Lee catchment; ^(d)^ Value for Hg and its compounds. Text in italics and bold indicates sampling points on tributaries.

**Table 3:** Overview of the range of concentrations (minimum-maximum) and mean (µg g^−1^ ± SD) total metal concentrations in surficial sediments determined at five sampling sites (*n* = 8) together with relevant sediment quality guidelines.

Site	Cd		Cu		Hg		Ni		Pb		Sn		Zn	
	Range	Mean ± SD	Range	Mean ± SD	Range	Mean ± SD	Range	Mean ± SD	Range	Mean ± SD	Range	Mean ± SD	Range	Mean ± SD
A	0.47–0.88	0.67 ± 0.20	32.6–73.7	46.1 ± 13.3	0.01–1.02	0.19 ± 0.30	1.34–19.5	13.7 ± 6.60	50.8–88.8	68.2 ± 11.4	0.67–7.98	4.66 ± 3.00	109–219	164 ± 41.6
***B***	***0.48–3.15***	***1.20*** ***±*** ***0.90***	***40.3–150***	***79.6*** ***±*** ***41.9***	***0.01–1.68***	***0.53*** ***±*** ***0.60***	***0.01–16.2***	***5.91*** ***±*** ***5.80***	***105–241***	***171*** ***±*** ***50.6***	***1.36–73.9***	***21.4*** ***±*** ***24.1***	***170–606***	***373*** ***±*** ***169***
***C***	***0.18–1.21***	***0.45*** ***±*** ***0.30***	***35.6–294***	***91.8*** ***±*** ***91.1***	***0.01–1.32***	***0.40*** ***±*** ***0.51***	***0.14–18.9***	***8.88*** ***±*** ***5.93***	***50.0–253***	***96.2*** ***±*** ***68.4***	***0.55–27.6***	***13.9*** ***±*** ***10.3***	***94.1–617***	***231*** ***±*** ***164***
D	1.90–2.61	2.28 ± 0.30	128–259	208 ± 43.4	0.05–1.02	0.58 ± 0.30	18.5–32.4	26.6 ± 4.30	186–322	265 ± 45.6	3.71–42.8	28.4 ± 14.6	549–946	777 ± 152
E	3.72–5.64	5.01 ± 0.60	108–161	127 ± 16.3	0.49–1.57	1.07 ± 0.40	29.0–51.1	38.2 ± 6.20	169–213	197 ± 12.9	3.30–23.1	15.9 ± 6.50	535–665	605 ± 48.9
Dutch TV ^1^	0.80		36.0		0.30		35.0		85.0				140	
Dutch IV ^2^	12.0		190		10.0		210		530				720	
Canadian ISQG ^3^	0.60		35.7		0.17				35.0				123	
Canadian PEL ^4^	3.50		197		0.48				91.3				315	

^1^ Target value—indicate the level that has to be achieved to fully recover the functional properties of the soil/sediment for humans, plant and animal life; ^2^ Intervention value—indicate when the functional properties of the soil/sediment for humans, plant and animal life, is seriously impaired or threatened; ^3^ Interim sediment quality guideline—represents the concentration below which adverse biological effects are expected to occur rarely; ^4^ Probable effect level—defines the level above which adverse biological effects are expected to occur frequently. Text in italics and bold indicates sampling points on tributaries.

**Table 4:** Mass percentage of metals released from sediments collected at five different sites into overlying water in laboratory-based batch tests over a 24 h mixing period.

Site	Cd	Cu	Hg	Ni	Pb	Sn	Zn
A	3.33 ± 0.36	3.12 ± 0.04	0.64 ± 1.93	3.15 ± 0.39	0.89 ± 0.14	0.12 ± 0.11	3.17 ± 0.02
B	6.12 ± 1.25	4.39 ± 0.01	0.33 ± 2.67	4.47 ± 0.16	1.03 ± 0.01	0.50 ± 0.08	4.73 ± 0.10
C	4.00 ± 0.67	1.95 ± 0.12	0.33 ± 1.00	1.99 ± 0.25	0.52 ± 0.05	0.14 ± 0.03	3.12 ± 0.05
D	4.97 ± 0.26	2.26 ± 0.12	0.42 ± 0.49	3.58 ± 0.10	0.90 ± 0.00	0.33 ± 0.08	3.82 ± 0.01
E	3.84 ± 0.11	2.85 ± 0.04	0.27 ± 0.29	3.28 ± 0.05	0.66 ± 0.01	0.26 ± 0.02	3.33 ± 0.01
